# Genomic information of *Kocuria* isolates from sake brewing process

**DOI:** 10.3934/microbiol.2021008

**Published:** 2021-02-26

**Authors:** Momoka Terasaki, Yukiko Kimura, Masato Yamada, Hiromi Nishida

**Affiliations:** 1 Biotechnology Research Center and Department of Biotechnology, Toyama Prefectural University, 5180 Kurokawa, Imizu, Toyama 939-0398, Japan; 2 Narimasa Sake Brewery, 418 Tachi, Nanto, Toyama 939-1676, Japan

**Keywords:** bacterial genome, chromosome, horizontal transfer, *Kocuria*, plasmid, sake brewing, transposon

## Abstract

Bacteria belonging to the genus *Kocuria* were identified as bacteria peculiar to a sake brewery in Toyama, Japan. Comparison of the 16S rRNA gene sequences revealed two groups of *Kocuria* isolates. Among known species, one group was similar to *K. koreensis* (*Kk* type), and the other, *K. uropygioeca* (*Ku* type). We determined complete genomic DNA sequences from two isolates, TGY1120_3 and TGY1127_2, which belong to types *Kk* and *Ku*, respectively. Comparison of these genomic information showed that these isolates differ at the species level with different genomic characters. Isolate TGY1120_3 comprised one chromosome and three plasmids, and the same transposon coding region was located on two loci on the chromosome and one locus on one plasmid, suggesting that the genetic element may be transferred between the chromosome and plasmid. Isolate TGY1127_2 comprised one chromosome and one plasmid. This plasmid encoded an identical transposase coding region, strongly suggesting that the genetic element may be transferred between these different isolates through plasmids. These four plasmids carried a highly similar region, indicating that they share a common ancestor. Thus, these two isolates may form a community and exchange their genetic information during sake brewing.

## 1. Introduction

Sake is a traditional Japanese alcoholic beverage. During sake brewing, the filamentous fungus *Aspergillus oryzae* converts rice starch into sugars; then, the yeast *Saccharomyces cerevisiae* converts the sugars into ethanol [1]. Sake brewing is not performed under completely sterile conditions. Therefore, numerous microorganisms are present at the beginning of the production process. As this process progresses, it gradually becomes suitable for yeast growth; eventually, no microorganisms but yeast persist due to their production of high ethanol concentrations (approximately 20%).

In the past, sake was produced using different *S. cerevisiae* strains that inhabited each sake brewery, known as *kuratsuki* yeast [2],[3]. The sake yeast unique to each sake brewery thus influenced production. Currently, many sake breweries produce sake using some selected sake yeasts (*Kyokai* yeasts) that are managed by the Brewing Society of Japan (*Jozo-kyokai*) [3],[4]. However, these yeasts were once established in specific sake breweries [3].

*A. oryzae* and *S. cerevisiae* are essential for sake production. As additional microorganisms are not added during the production process, any bacterial DNA detected in sake products must come from the bacteria entered during the production process [5],[6]. Highly ethanol-tolerant lactic acid bacteria can spoil sake [7]. This spoilage is prevented using pasteurization, which has been performed since the 16th century in Japan, before Louis Pasteur's invention.

Comparative studies of bacterial DNA sequences in sake have detected diverse types of bacterial DNA, indicating that various bacteria grew during the sake production process [5],[8]–[12]. Although bacterial DNA is detected in sake products, most bacteria die due to high ethanol concentrations. Thus, no bacteria were isolated from sake products. Therefore, we isolated bacteria from samples of the first mixture (*Hatsuzoe*) of *koji* (steamed rice covered with *A. oryzae*) and *moto* (fermentation starter including *S. cerevisiae*) and then identified the isolates. We obtained 46 bacterial isolates from six different *Hatsuzoe* samples in Brewery Toyama 1 [12].

In our previous study, DNA of the genus *Kocuria* was detected only in cloudy sake and *sake-kasu* (sake lees) of Brewery Toyama 1 [12], strongly suggesting that *Kocuria* may be specific to this sake brewery. In addition, *Kocuria* DNA was not detected in clear sake from Brewery Toyama 1 [12], indicating that *Kocuria* cells may be difficult to lyse during sake production. This is consistent with the fact that ethanol does not disrupt *Kocuria rhizophila* cells [13].

In this study, genomic DNA of the *Kocuria* isolates from *Hatsuzoe* of the sake brewery were sequenced and compared.

## 2. Methods

### 2.1. Culturing, DNA isolation, and PCR

YPD (20 g/L tryptone, 10 g/L yeast extract, 2.0% glucose) medium and YPD agar plate were used for culturing each isolate. Each isolate was incubated overnight at 37 °C or for 2 days at 30 °C. DNA of each isolate was extracted from the culture (1 mL) and purified using NucleoSpin Tissue (Macherey-Nagel, Düren, Germany). The V3–V4 region of the 16S rRNA gene from each DNA were amplified using the PCR primers, 5′-ATGTGTATAAGAGACAGCCTACGGGNGGCWGCAG-3′ and 5′-TGTATAAGAGACAGGACTACHVGGGTATCTAATCC-3′, which were designed by modification of method of Illumina, San Diego, USA. The PCR products were sequenced using the primer 5′-TGTATAAGAGACAGGACTAC-3′.

### 2.2. Phylogenetic analysis

A neighbor-joining tree was constructed with 1000 bootstrap replicates using MEGA X [14]. The evolutionary distances were computed using the Maximum Composite Likelihood method [15] and presented in units of the number of base substitutions per site. This analysis involved 14 nucleotide sequences of 16S rRNA gene. All positions containing gaps and missing data were eliminated (complete deletion option). In total, the final 16S rRNA gene dataset revealed 1407 positions.

### 2.3. Genome sequencing

The genomes of isolates TGY1120_3 and TGY1127_2 were determined using both Nanopore (GridION X5; Oxford, UK) and Illumina (MiSeq; San Diego, CA, USA) DNA sequencers. The hybrid assembly of the two sequence data was performed using Unicycler version 0.4.7 [16] or MaSuRCA version 3.3.3 [17]. Circulation analysis was performed using Circlator [18]. Illumina short reads mapping was performed using BWA version 0.7.17 [19], and then error correction was performed using Pilon version 1.23 [20].

## 3. Results and discussion

### 3.1. Isolation of Kocuria living in a sake brewery

In our previous paper [12], we found 46 bacterial isolates from 6 different *Hatsuzoe* samples from Brewery Toyama 1. Based on the partial 16S rRNA gene sequences, 23, 12, 6, 2, 2, and 1 isolates from 6, 4, 3, 1, 1, and 1 *Hatsuzoe* samples had similar sequences to the genera *Kocuria*, *Staphylococcus*, *Bacillus*, *Leifsonia*, *Microbacterium*, and *Enterococcus*, respectively [12]. Thus, these genera belong to gram-positive bacteria.

*Kocuria* isolates were obtained from all 6 *Hatsuzoe* samples [12]. In addition, DNA from the genus *Kocuria* was detected in cloudy sake and *sake-kasu* of the same sake brewery (Brewery Toyama 1), but was not detected in the other breweries [12]. Thus, these *Kocuria* isolates probably inhabit the sake brewery.

In the traditional sake brewing, non-spoilage lactic acid bacteria that inhabit a sake brewery have been used for preventing growth of other bacteria with lactic acid [9]. *Kocuria* is a gram-positive coccus, which belongs to actinomycetes [21]. To our knowledge, this is the first report on the isolation of bacteria other than lactic acid bacteria living in a sake brewery.

### 3.2. Detection of different types of Kocuria

Comparison of the partial 16S rRNA gene sequences revealed two groups of *Kocuria* isolates (Supplementary File 1). Among 23 *Kocuria* isolates, the sequences of 5 isolates were identical to the sequence of ***K. koreensis*,** and those of 18 isolates were identical to that of *K. uropygioeca*. The species of *K. koreensis* and *K. uropygioeca* are evolutionarily closely related [22],[23]. Here, we termed the 5 and 18 isolates as *Kk* and *Ku* types, respectively (Table 1). In *Hatsuzoe* samples 3, 4, and 6, both types were detected (Table 1), indicating that different types of *Kocuria* lived in the *Hatsuzoe*.

**Table 1.** *Kocuria* isolates from *Hatsuzoe* samples.

*Hatsuzoe*	Sampling date	Isolate	16S rRNA type
Sample 1	October 23, 2018	LB1023_2	*Ku*
		TGY1023_2	*Ku*
Sample 2	November 13, 2018	LB1113_2	*Ku*
		MB1113_2	*Ku*
		TGY1113_2	*Ku*
Sample 3	November 20, 2018	LB1120_1_30	*Ku*
		LB1120_2	*Kk*
		MB1120_1	*Ku*
		TGY1120_1	*Ku*
		TGY1120_3	*Kk*
Sample 4	November 27, 2018	LB1127_2	*Ku*
		LB1127_3	*Kk*
		MB1127_2	*Ku*
		TGY1127_2	*Ku*
Sample 5	December 4, 2018	LB1204_1	*Ku*
Sample 6	December 18, 2018	LB1218_1_15	*Ku*
		LB1218_1_30	*Ku*
		LB1218_2_30	*Kk*
		MB1218_1_15	*Ku*
		MB1218_2_30	*Ku*
		MB1218_5_30	*Kk*
		TGY1218_1_15	*Ku*
		TGY1218_1_30	*Ku*

*Kocuria* is a microorganism detected in fermented foods and beverages [24]. These isolates can grow in not only the general bacterial media such as Luria-Bertani and Tryptone, Glucose, and Yeast extract (TGY), but also in marine broth used for culturing marine bacteria [12]. This suggests that these isolates can grow in various environments, including that of the sake production process.

### 3.3. Kocuria isolates differ at the species level with different genomic characters

We selected randomly two isolates from each type and performed whole-genome DNA sequencing of isolate TGY1120_3 belonging to *Kk* type and isolate TGY1127_2 belonging to *Ku* type. Isolate TGY1120_3 had one chromosome (Accession number, AP022830) and three plasmids, pTGY1120_3_1 (AP022831), pTGY1120_3_2 (AP022832), and pTGY1120_3_3 (AP022833); and isolate TGY1127_2 had one chromosome (AP022834) and one plasmid, pTGY1127_2_1 (AP022835) (Table 2). The GC content of each plasmid was lower than that of the host chromosome (Table 2). This bias of GC content is commonly observed between bacterial plasmids and their host chromosomes [25],[26]. Based on gene annotation, isolate TGY1120_3 had 2556 protein-coding genes (including 1036 hypothetical proteins), 48 tRNA-coding genes, and 9 rRNA-coding genes (Table S1). Isolate TGY1127_2 had 2648 protein-coding genes (including 1085 hypothetical proteins), 50 tRNA-coding genes, and 9 rRNA-coding genes (Table S2).

**Table 2.** DNA size and GC content of chromosomes and plasmids of *Kocuria* isolates.

	Size (nucleotides)	GC content (%)
Chromosome of isolate TGY1120_3	2,838,001	64.8
pTGY1120_3_1	32,375	59.6
pTGY1120_3_2	32,277	58.7
pTGY1120_3_3	27,385	58.6
Chromosome of isolate TGY1127_2	2,963,611	60.9
pTGY1127_2_1	33,410	60.3

**Figure 1.**
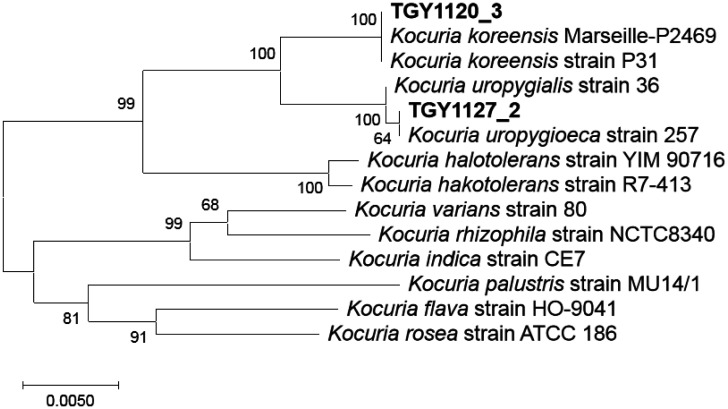
Phylogenetic relationships among 16S rRNA gene sequences of the two isolates and related organisms. The neighbor-joining tree was constructed with 1000 bootstrap replicates using MEGA X [14]. The evolutionary distances were computed using the Maximum Composite Likelihood method [15]. All positions containing gaps and missing data were eliminated (complete deletion option). In total, the final dataset contained 1407 positions. The bar indicates a 0.5% difference.

Based on the phylogenetic tree of complete 16S rRNA gene sequences, isolate TGY1120_3 belongs to *K. koreensis* and isolate TGY1127_2 belongs to *K. uropygioeca*, which is consistent with the partial 16S rRNA gene sequence comparison (Figure 1). Thus, these isolates differ at the species level.

Not only 16S rRNA gene but also other genomic information differs between TGY1120_3 and TGY1127_2. The genomic information showed that TGY1120_3 and TGY1127_2 have 4 (TGY1120_3_00327, TGY1120_3_02298, TGY1120_3_02384, and TGY1120_3_02479, see Table S1) and 7 (TGY1127_2_00485, TGY1127_2_01264, TGY1127_2_01480, TGY1127_2_01984, TGY1127_2_02447, TGY1127_2_02542, and TGY1127_2_02644, see Table S2) alcohol dehydrogenase homologs. Alcohol dehydrogenase is reported to be associated with ethanol resistance [27],[28]. In addition, although TGY1127_2 has urease complex homologs (gene cluster TGY1127_2_01020 to TGY1127_2_01028, see Table S2), TGY1120_3 lacks them. Urease is reported to be associated with acid resistance [29]–[32]. It strongly suggests that TGY1127_2 has stronger acid and alcohol resistance than TGY1120_3.

### 3.4. Distribution of identical transposase genes in Kocuria isolates

**Figure 2.**
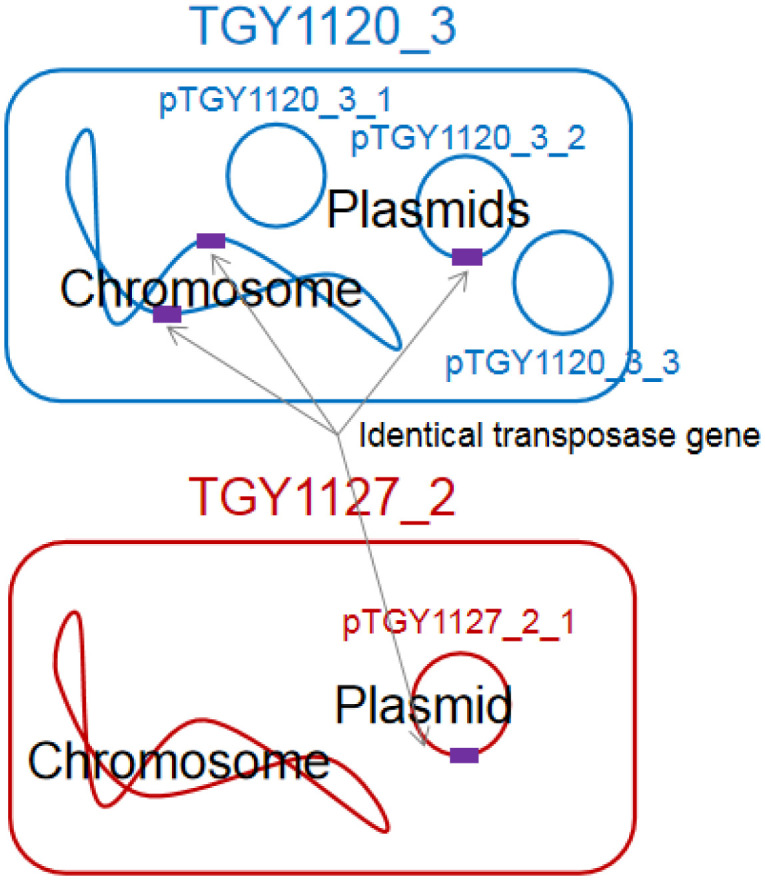
Distribution of identical transposase gene encoding ISL3 family transposase ISAar30 among *Kocuria* isolates TGY1123_3 and TGY1127_2.

Isolate TGY1120_3 had two ISL3 family transposase ISAar30 coding genes (TGY1120_3_00129 and TGY1120_3_00291, see Table S1) on different loci of the chromosome and one (TGY1120_3_02579, see Table S1) on the plasmid pTGY1120_3_2 (Figure 2). The DNA length was 1308 nt, encoding 435 amino acids. These three DNA sequences were identical, strongly suggesting that these regions were recently transferred between the chromosome and plasmid of *Kocuria* isolate TGY1120_3. The GC content of the ISL3 family transposase ISAar30 cording gene is 69.3%, which is higher than those of the chromosome (64.8%) and the plasmid pTGY1120_3_2 (58.7%) (Table 2). Bacteria have the nucleoid-associated proteins that bind horizontally transferred DNA regions with low GC content rather than the remaining DNA and inhibit expression from those regions [33],[34]. However, to our knowledge, there are no reports of proteins that bind to regions with high GC content and inhibit gene expression. It suggests that this gene may be transferred from bacteria with high GC (approximately 70%) content genome and be active in isolate TGY1120_3. Surprisingly, an identical ISL3 family transposase ISAar30 coding gene (TGY1127_2_02700, see Table S2) also existed on the plasmid pTGY1127_2_1 of isolate TGY1127_2 (Figure 2), strongly indicating that this transposon coding gene was recently transferred between these two *Kocuria* isolates. To confirm this, it is necessary to show the horizontal transfer of the plasmids between different isolates and the transfer of the transposons in each cell experimentally.

The Basic Local Alignment Search Tool (BLAST) on the National Center for Biotechnology Information website was used to identify this transposase or its transposase coding region in the international DNA database. The BLAST was performed using the default values. We found no identical 1308-nt DNA sequences, but sequences differing by 1 nt were found in *Acidipropionibacterium jensenii* FAM 19038 (genomic GC content: 68.7%) and *Propionibacterium freudenreichii* PFRJS17 (genomic GC content: 67.3%), and a sequence with a 2-nt difference was found in *Kocuria palustris* MU14/1 (genomic GC content: 70.5%). Although these three genera belong to Actinobacteria, *Acidipropionibacterium* and *Propionibacterium* belong to Propionibacteriales whereas *Kocuria* belongs to Micrococcales, which are not closely evolutionarily related [35]. *Acidipropionibacterium* and *Propionibacterium* were not found in any samples from Brewery Toyama 1 [12]. This strongly suggests that this transposable element may be transferred between different bacterial species (genera) in the natural environment.

In isolate TGY1120_3, the other differing transposase coding regions were located on the chromosome (one locus), pTGY1120_3_2 (two loci), and pTGY1120_3_3 (one locus) (Table S1). However, in isolate TGY1127_2, there was no other transposase (Table S2).

### 3.5. Plasmids have a common ancestor

**Figure 3.**
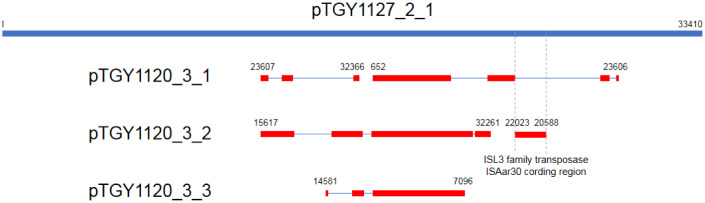
Graphic summary of the BLASTn results using the DNA sequence of pTGY1127_2_1 as the query. Red regions indicate high similar regions. Numbers indicate the position at each plasmid DNA sequence.

The three plasmids of isolate TGY1120_3 and the single plasmid of isolate TGY1127_2 had similar DNA sequences (Figure 3), suggesting that these four plasmids share a common ancestor. Thus, plasmid variation occurred in different *Kocuria* species after common plasmid acquisition. If so, the ISL3 family transposase ISAar30 coding region may exist on the common plasmid. Further research is needed to elucidate the relationships between the plasmids and transposable genetic elements.

In a sake brewing, ethanol produced by sake yeast kills bacteria. Therefore, bacteria may develop a survival strategy. Our findings suggest that *Kocuria* genome evolution may occur using plasmids and transposons in sake brewing environments.

## 4. Conclusions

This is the first report of bacteria other than lactic acid bacteria peculiar to a sake brewery (*kuratsuki* bacteria), which were isolated and subjected to genomic DNA sequencing. Our findings show that the *kuratsuki* bacterial isolates are not one strain of *Kocuria*, but several strains, which may form a community and exchange their genetic information between different isolates during the sake production process. Plasmids play an important role in the genetic diversity of *Kocuria*, suggesting that natural gene manipulation may have occurred in the sake tank during sake production.

## References

[1] Kitagaki H, Kitamoto K (2013). Breeding research on sake yeasts in Japan: history, recent technological advances, and future perspectives. Ann Rev Food Sci Technol.

[2] Akao T, Yashiro I, Hosoyama A (2011). Whole-genome sequencing of sake yeast *Saccharomyces cerevisiae* Kyokai no. 7. DNA Res.

[3] Ohya Y, Kashima M (2019). History, lineage and phenotypic differentiation of sake yeast. Biosci Biotechnol Biochem.

[4] Azumi M, Goto-Yamamoto N (2001). AFLP analysis of type strains and laboratory and industrial strains of *Saccharomyces cerevisiae stricto* and its application to phenetic clustering. Yeast.

[5] Terasaki M, Fukuyama A, Takahashi Y (2017). Bacterial DNA detected in Japanese rice wines and the fermentation starters. Curr Microbiol.

[6] Akaike M, Miyagawa H, Kimura Y (2020). Chemical and bacterial components in sake and sake production process. Curr Microbiol.

[7] Suzuki K, Asano S, Iijima K (2008). Sake and beer spoilage lactic acid bacteria—a review. J Inst Brew.

[8] Bokulich NA, Ohta M, Lee M (2014). Indigenous bacteria and fungi drive traditional kimoto sake fermentations. Appl Environ Microbiol.

[9] Koyanagi T, Nakagawa A, Kiyohara M (2016). Tracing microbiota changes in *yamahai-moto*, the traditional Japanese sake starter. Biosci Biotech Biochem.

[10] Tsuji A, Kozawa M, Tokuda K (2018). Robust domination of *Lactobacillus sakei* in microbiota during traditional Japanese sake starter *yamahai-moto* fermentation and the accompanying changes in metabolites. Curr Microbiol.

[11] Terasaki M, Miyagawa S, Yamada M (2018). Detection of bacterial DNA during the process of sake production using *sokujo-moto*. Curr Microbiol.

[12] Terasaki M, Nishida H (2020). Bacterial DNA diversity among clear and cloudy sakes, and *sake-kasu*. Open Bioinfo J.

[13] Fujita K, Hagishita T, Kurita S (2006). The cell structural properties of *Kocuria rhizophila* for aliphatic alcohol exposure. Enzyme Microbial Technol.

[14] Kumar S, Stecher G, Li M (2018). MEGA X: Molecular Evolutionary Genetics Analysis across computing platforms. Mol Biol Evol.

[15] Tamura K, Nei M, Kumar S (2004). Prospects for inferring very large phylogenies by using the neighbor-joining method. Proc Natl Acad Sci USA.

[16] Wick RR, Judd LM, Gorrie CL (2017). Unicycler: resolving bacterial genome assemblies from short and long sequencing reads. Plos Comput Biol.

[17] Zimin AV, Marçais G, Puiu D (2013). The MaSuRCA genome assembler. Bioinformatics.

[18] Hunt M, De Silva N, Otto TD (2015). Circlator: automated circularization of genome assemblies using long sequencing reads. Genome Biol.

[19] Li H, Durbin R (2009). Fast and accurate short read alignment with Burrows-Wheeler Transform. Bioinformatics.

[20] Walker BJ, Abeel T, Shea T (2014). Pilon: An integrated tool for comprehensive microbial variant detection and genome assembly improvement. Plos One.

[21] Stackebrandt E, Koch C, Gvozdiak O (1995). Taxonomic dissection of the genus *Micrococcus*: *Kocuria* gen. nov., *Nesterenkonia* gen. nov., *Kytococcus* gen. nov., *Dermacoccus* gen. nov., and *Micrococcus* Cohn 1872 gen. emend. Int J Syst Evol Microbiol.

[22] Braun MS, Wang E, Zimmermann S (2018). *Kocuria uropygioeca* sp. nov. and *Kocuria uropygialis* sp. nov., isolated from the preen glands of Great Spotted Woodpeckers (*Dendrocopos major*). Syst Appl Microbiol.

[23] Park E-J, Roh SW, Kim M-S (2010). *Kocuria koreensis* sp. nov., isolated from fermented seafood. Int J Syst Evol Microbiol.

[24] Tamang JP, Watanabe K, Holzapfel WH (2016). Review: Diversity of microorganisms in global fermented foods and beverages. Front Microbiol.

[25] Rocha EPC, Danchin A (2002). Base composition bias might result from competition for metabolic resources. Trends Genet.

[26] Nishida H (2012). Comparative analyses of base compositions, DNA sizes, and dinucleotide frequency profiles in archaeal and bacterial chromosomes and plasmids. Int J Evol Biol.

[27] Zheng Y, Zhang K, Su G (2015). The evolutionary response of alcohol dehydrogenase and aldehyde dehydrogenase of *Acetobacter pasteurianus* CGMCC 3089 to ethanol adaptation. Food Sci Biotechnol.

[28] Luong TT, Kim E-H, Bak JP (2015). Ethanol-induced alcohol dehydrogenase E (AdhE) potentiates pneumolysin in *Streptococcus pneumoniae*. Infect Immun.

[29] De Koning-Ward TF, Robins-Browne RM (1995). Contribution of urease to acid tolerance in *Yersinia enterocolitica*. Infect Immun.

[30] Weeks DL, Eskandari S, Scott DR (2000). A H^+^-gated urea channel: the link between *Helicobacter pylori* urease and gastric colonization. Science.

[31] Maroncle N, Rich C, Forestier C (2006). The role of *Klebsiella pneumoniae* urease in intestinal colonization and resistance to gastrointestinal stress. Res Microbiol.

[32] Sangari FJ, Seoane A, Rodríguez MC (2007). Characterization of the urease operon of *Brucella abortus* and assessment of its role in virulence of the bacterium. Infect Immun.

[33] Navarre WW, Porwollik S, Wang Y (2006). Selective silencing of foreign DNA with low GC content by the H-NS protein in *Salmonella*. Science.

[34] Nishida H (2013). Genome DNA sequence variation, evolution, and function in bacteria and archaea. Curr Issues Mol Biol.

[35] Gao B, Gupta RS (2012). Phylogenetic framework and molecular signatures for the main clades of the phylum *Actinobacteria*. Microbiol Mol Biol Rev.

